# AtPiezo Plays an Important Role in Root Cap Mechanotransduction

**DOI:** 10.3390/ijms22010467

**Published:** 2021-01-05

**Authors:** Xianming Fang, Beibei Liu, Qianshuo Shao, Xuemei Huang, Jia Li, Sheng Luan, Kai He

**Affiliations:** 1 Ministry of Education Key Laboratory of Cell Activities and Stress Adaptations, School of Life Sciences, Lanzhou University, Lanzhou 730000, China; fangxm18@lzu.edu.cn (X.F.); liubb19@lzu.edu.cn (B.L.); shaoqsh19@lzu.edu.cn (Q.S.); huangxm17@lzu.edu.cn (X.H.); lijia@lzu.edu.cn (J.L.); 2 Department of Plant and Microbial Biology, University of California, Berkeley, CA 94720, USA

**Keywords:** *Arabidopsis*, mechanical stimuli, mechanosensitive ion channel, AtPiezo, root cap, Ca^2+^

## Abstract

Plants encounter a variety of mechanical stimuli during their growth and development. It is currently believed that mechanosensitive ion channels play an essential role in the initial perception of mechanical force in plants. Over the past decade, the study of Piezo, a mechanosensitive ion channel in animals, has made significant progress. It has been proved that the perception of mechanical force in various physiological processes of animals is indispensable. However, little is still known about the function of its homologs in plants. In this study, by investigating the function of the *AtPiezo* gene in the model plant *Arabidopsis thaliana*, we found that *AtPiezo* plays a role in the perception of mechanical force in plant root cap and the flow of Ca^2+^ is involved in this process. These findings allow us to understand the function of AtPiezo from the perspective of plants and provide new insights into the mechanism of plant root cap in response to mechanical stimuli.

## 1. Introduction

As sessile organisms, most plants are subjected to various stresses in the environment. Among the complex environmental cues, mechanical stimuli are the important factors including gravity, touch, winding, raining, wounding, feeding, obstacles, etc., which affect the growth and development of plants [1,2]. In order to adapt to the environment, plants have evolved various mechanisms for sensing and responding to mechanical stimuli. Gravity can change the position of amyloplasts in the columella cells and endodermal cells. The signal of the physical change of position eventually alters the concentration of auxin in different parts, causing the plant roots to grow towards gravity direction [3,4,5]. *Arabidopsis* will delay its flowering time after being touched regularly. The term thigmomorphogenesis is used to describe the mechanically induced physiological and morphological responses of plants [6]. A few touch-induced genes have been discovered. These genes are mainly encoding proteins related to calcium signals, enzymes related to pectin and cellulose biosynthesis, and kinases involved in plant disease resistance [6,7]. A recent report showed that TRHEP1, a cytoplasmic protein that can be phosphorylated, is involved in the process of flowering delay in response to touch treatment [8].

One of the ways that plants respond to mechanical stimuli is that the mechanical force causes the cell membrane to deform [9]. There are reports showing that some receptor kinases connected to the cell wall may be involved in the perception of mechanical force [10,11]. In addition, a plausible hypothesis is that the mechanosensitive ion channels anchored on the membrane can change their structural conformation in response to the shape alteration of the cell membrane. The activity of the ion channel causes ion flow, which further affects downstream intracellular events [9]. To date, only a few of the mechanosensitive ion channels have been functionally analyzed in plants, including MSLs, MCA, TPK and OSCA1. Plant *MSL* family is a group of genes homologous to *MscS* gene, which is related to cell turgor regulation in *Escherichia coli*. There are 10 *MscS* homolog genes in *Arabidopsis*. MSL8 was shown to be involved in pollen turgor regulation during pollen hydration [12]. MSL10 is involved in plant root cell swelling [13]. *MCA1* is a plant-unique gene. It has been proved to be a tension-activated calcium ion channel. The roots of *MCA1* loos-of-function mutant show impaired ability to penetrate in the agar media [14]. Recent studies have shown that MCA1 is also involved in plant response to gravity [15]. The *TPK* gene in plants is homologous to the *TPK* family genes in animals. TPK is mainly located to the vacuolar membrane. It mediates large quantity of potassium ions under the conditions of changing membrane tension although it has channel activity under normal conditions [16]. OSCA1 was originally found to be involved in osmotic sensation in plants. Later work has shown that the OSCA family is homologous to the animal TMEM63 family and functions as mechanosensitive ion channels [17,18].

For most plants, it is important to forage into the soil for stable growth and obtaining nutrients from the environment. Changes in mechanical force represented by soil structure and moisture content have significant impact on the formation of root systems [19]. The roots keep growing to pass through the objects when encountering mechanical obstacles. Or, when the roots cannot pass through the obstacles, the different growth direction is taken [10]. When the roots travel through the soil, the pressure on the root tip is the most important because the root tip is the primary area where the plant contacts the soil [20,21]. The root cap, as the outermost part of the root tip, plays a key role in its response to soil mechanical forces [22]. The important functions of the root cap are to protect the root tip from the impact of the environment, to determine the direction of root growth, and to reduce the friction between the soil by shedding surrounding root cap cells and secreting mucus [23,24]. Previous studies have shown that the soil status causes significant changes in root cap morphology [21,25].

Piezo1 and Piezo2 are mechanically sensitive ion channels originally found in mouse [26]. In the following 10 years, studies have shown that animal Piezo plays an essential role in light sensing, touching, proprioception, vascular blood flow regulation, differentiation of stem cells, and proliferation of the stretch-induced cell [27,28,29,30,31,32]. The studies of the protein structure revealed its working model of forming ion channel in the form of heterotrimers [33,34]. *MmPiezo1* encodes a membrane protein with 38 transmembrane helixes, and its homologous genes are widely presented in animals and plants [26,34]. Most plants only have one gene homologous to animal *Piezo* [35]. In *Arabidopsis*, recent studies have shown that it regulates the spread of viruses in plants, with no report of its function in mechanical force transduction [35]. In this study, we analyzed the functions of *AtPiezo* in the model plant *Arabidopsis*. We found that *AtPiezo* has a specific expression in the root cap. By using genetic and physiological approaches, we show that AtPiezo likely plays an important role in plant root cap for the perception of mechanical force.

## 2. Results

### 2.1. Identification and Analysis of Piezo Genes in Plants

**Figure 1:**
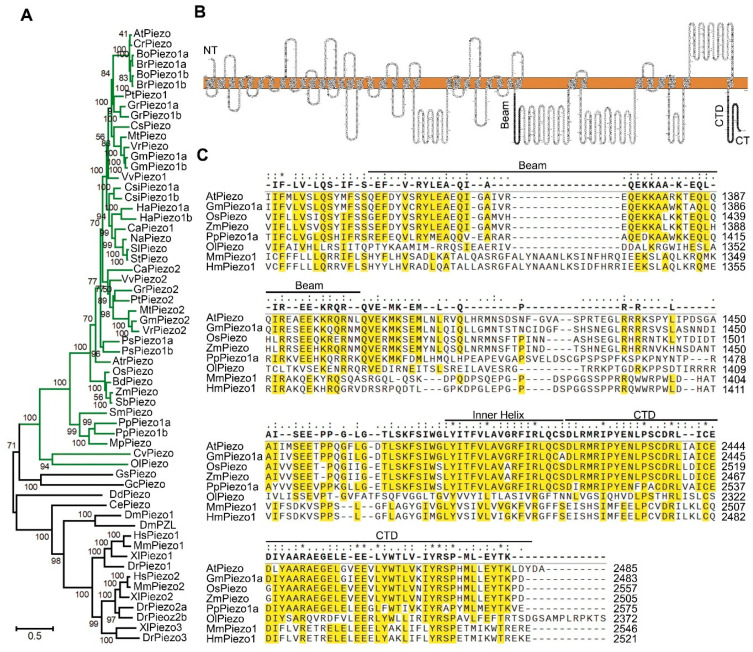
Evolutionary Analysis of Piezo in Plants. (**A**) Maximum likelihood unrooted phylogenetic tree of Piezo homologs. Green branches belong to the plant kingdom. (**B**) Predicted topology of the AtPiezo monomer. Beam and CTD domains are indicated in black. (**C**) The alignment of beam, inner helix and CTD domains of AtPiezo and its orthologs. Protein multiple sequence alignment of AtPiezo (*Arabidopsis thaliana*), GmPiezo1a (*Glycine max*), OsPiezo (*Oryza sativa Japonica Group*), ZmPiezo (*Zea mays*), PpPiezo1a (*Physcomitrella patens*), OlPiezo (*Ostreococcus lucimarinus*), MmPiezo1 (*Mus musculus*), and HmPiezo1 (*Homo sapiens*). The highly conserved amino acids among Piezo orthologues are highlighted in yellow. The consensus is with a threshold of >50%.

In order to analyze the evolutionary relationship of the *Piezo* genes in plants, we searched the NCBI database for the *Piezo* homologous genes to the mouse *MmPiezo1*. We selected 37 representative species for evolutionary analysis, 30 of which belong to the plant kingdom (Table S1). Both Maximum Likelihood tree (Figure 1A) and Neighbor-Joining (Figure S1A) tree based on full protein sequences showed similar results. *Piezo* genes in all species may be derived from a single ancestral gene, and then gene duplication and loss events occurred along with the evolutionary process. Most plants retain 1 to 3 *Piezo* genes (Figure S1B). There is only one *Piezo* gene in basal land plants and monocots except for *Physcomitrella patens*. In other flowering plants, three *Piezo* homologous genes were found in *Glycine max* and *Gossypium raimondii*. There are two copies of the *Piezo* genes in *Vitis vinifera*, *Helianthus annuus* and other species and only one *Piezo* homologous gene in *Arabidopsis thaliana*. It may have lost a copy during evolution in *Arabidopsis* since other species retain two copies in *Cruciferae* (Figure 1A). Protein sequence alignment result indicated that *AtPiezo* has 15.54% identity to *MmPiezo1*. According to the prediction of protein structure, AtPiezo contains at least 31 transmembrane domains (Figure 1B). The study of the MmPiezo1 protein structure revealed that its C-terminal (CT) structure of the pore determines ion-permeation properties [36]. The multiple protein sequence alignment of MmPiezo1, HmPiezo1 and its orthologs in other five species of plant kingdom indicated highly conserved regions at the C-terminal, including inner helix and C-terminal domain (CTD) (Figure 1C). The beam domain of the MmPiezo1 protein could transmit force from the periphery to the central ion channel [36]. However, the evolutionary conservation of beam domains is lower between plants and animals (Figure 1C). In summary, we found *Piezo* genes in almost all plant species and the C-terminal domains of Piezo proteins are conserved.

### 2.2. AtPiezo Is Expressed in Root Cap

**Figure 2:**
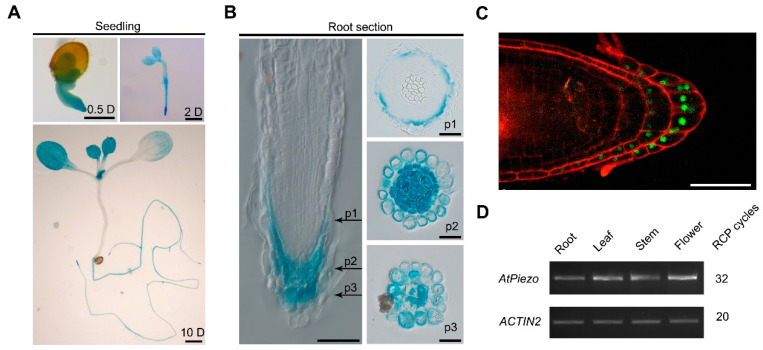
Expression patterns of *AtPiezo* in *Arabidopsis*. (**A**) Histochemical GUS staining is shown in 0.5-day-old, 2-day-old, and 10-day-old seedlings. Bars = 0.5 mm. (**B**) Longitudinal and cross sections of the root cap in different positions of *pAtPiezo::GUS* plant. Bars = 50 μm and 20 μm. (**C**) The expression of *pAtPiezo::NLS-YFP* in the root cap. Bars = 50 μm. (**D**) RT-PCR analysis of *AtPiezo* transcripts in different tissues.

To determine the expression patterns of *AtPiezo* in *Arabidopsis*, we cloned 1559 bp of *AtPiezo* promoter to drive the *GUS* reporter gene. In the 10-day-old transgenic seedlings, we found that *AtPiezo* is highly expressed in the root and young leaves (Figure 2A). In the root of the seedlings, *AtPiezo* is specifically expressed in the root vascular system and root tip (Figure 2A and Figure S2A). The cross-section of the root tip revealed that *AtPiezo* is mainly expressed in the root cap (Figure 2B). The *AtPiezo* native promoter-driven YFP that is nuclear-localized also showed a similar expression pattern (Figure 2C). We also detected the expression of *GUS* gene in guard cell, vascular tissue, and pollen (Figure S2B). Finally, the results of *AtPiezo* gene expression in different tissues and organs were confirmed by RT-PCR, which is consistent with the results of GUS staining (Figure 2D).

### 2.3. Atpiezo Mutants Exhibit Reduced Rooting Ability

**Figure 3:**
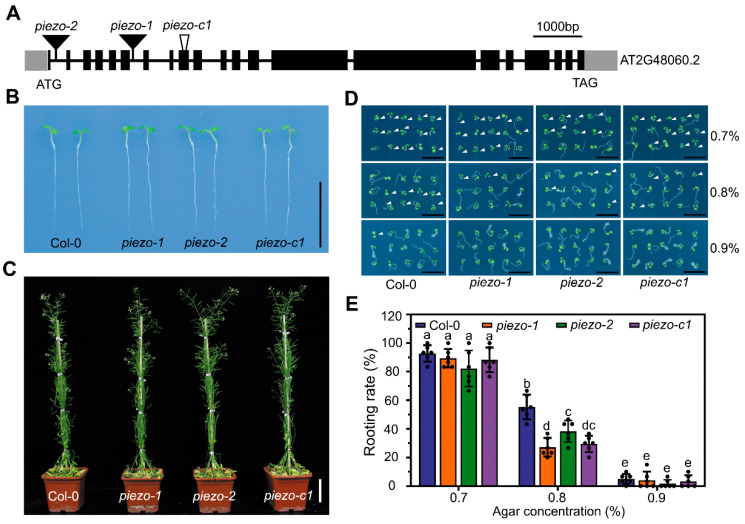
*atpiezo* mutants exhibit reduced rooting ability. (**A**) Schematic diagram of *AtPiezo* gene structure. Exons are indicated by black boxes. The black lines represent introns. Untranslated regions are indicated by gray boxes. The insertion sites of two T-DNA insertion alleles are indicated by the black solid triangles. The allele of delete fragment is indicated by the black hollow triangle. Bar = 1000 bp. (**B**) Representative images of WT and *atpiezo* mutants grown on 1/2MS medium five days after germination, Bar = 1 cm. (**C**) Representative images of WT and *atpiezo* mutants grown in the soil 55 days after germination. Bar = 5 cm. (**D**) The rooting phenotype of Col-0, *piezo-1*, *piezo-2 and piezo-c1* horizontally grown on the agar medium. Five-day-old seedlings of different plant lines were grown on the medium containing 0.7%, 0.8%, or 0.9% agar. White solid arrows indicate the seedlings rooting in the medium. Bar = 1 cm. (**E**) The rate of WT and *atpiezo* mutants foraging into the medium five days after germination. Data are presented as mean ± SD (*n* = 6 plates, more than 30 seedlings in each plate) and analyzed with two-way ANOVA and Tukey’s multiple comparison test (Different lowercase letters indicate significant differences at *p* < 0.05).

To investigate the functions of the *AtPiezo* gene, we ordered T-DNA insertion mutant lines *piezo-1* and *piezo-2*. The T-DNA of these two mutants is inserted into the introns of *AtPiezo* gene (Figure 3A and Figure S3A). By using RT-PCR, both lines were found to be null alleles (Figure S3B,C). We also used CRISPR/Cas9 approach to construct a mutant line *piezo-c1* with a 31bp-deletion in the seventh exon. The deletion of the fragment causes the premature termination of gene translation (Figure 3A and Figure S3D,E). We found that the *atpiezo* mutant plants did not differ from wild-type (WT) plants under normal growth conditions (Figure 3B,C). Due to the specific expression of *AtPiezo* in the root cap, we speculated that it may play a role in sensing mechanical force changes during root growth. To analyze plant rooting ability, we used the media containing different agar concentrations to mimic different levels of soil hardness. When the plants were grown on the media in a horizontally placed plate with 0.8% agar, the roots of *atpiezo* mutants showed decreased probability of entering medium compared to WT plants (Figure 3D). The results showed that the rooting rate of the *atpiezo* mutants decreased about 20% compared to WT (Figure 3E). The results indicated that AtPiezo may affect the response of plant root to the surface force of the medium.

### 2.4. Atpiezo Mutants Show Altered Growth Status Inside the Medium

**Figure 4:**
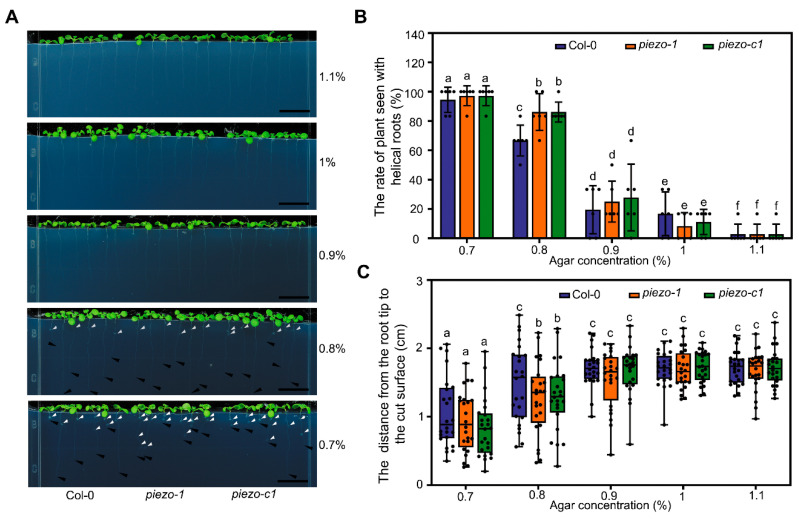
The *atpiezo* mutant plants show altered growth status inside the medium. (**A**) Representative images of growth status of primary roots of 7-day-old plants in the agar medium. White solid arrows indicate the helixes at primary roots. Black solid arrows indicate the positions where the root tips reach. Bar = 1 cm. (**B**) The rate of helical roots of WT and *atpiezo* mutants in the media with different agar concentrations. Data are presented as mean ± SD (*n* = 6 plates, more than 10 seedlings in each plate) and analyzed with two-way ANOVA and Tukey’s multiple comparison test (Different lowercase letters indicate significant differences at *p* < 0.05). (**C**) The distance from the root tip to the medium surface of WT and *atpiezo* mutants in the media with different agar concentrations. Boxplots span the first to the third quartiles of the data. A line in the box represents the median (*n* > 20). The results were analyzed with two-way ANOVA and Tukey’s multiple comparison test (Different lowercase letters indicate significant differences at *p* < 0.05).

To simulate the growth status of plants in the soil, we directly germinated the seeds within the medium and cultivated them vertically. We found that the plant roots can grow vertically in the medium with relatively higher concentration of agar; while the roots of the plants will appear helical roots when the agar concentration of the medium is lower (Figure 4A), which is consistent with the recently reported results [37]. We found that there was no difference between *atpiezo* mutants and WT when the concentration of agar was 0.7%, 0.9%, 1% or 1.1%. However, when the plants were grown in the medium with 0.8% agar, we observed increased helical roots in *atpiezo* mutants. Around 67% WT plants showed helical roots. Whereas, under the same condition, about 86% *atpiezo* mutant plants showed helical roots (Figure 4B). In addition, the depth of root tips reached in *atpiezo* mutants was shorter than that in WT when agar was 0.8% (Figure 4C). The average depth the roots of WT plants can reach was around 1.5 cm. By contrast, the average depth the roots of the *atpiezo* mutant plants can reach was around 1.2 cm. These results suggested that AtPiezo is involved in the response to the resistance from agar media (Figure 4C). Previous studies have shown that helical root can induce lateral root production [38].To further determine whether AtPiezo affects the root architecture, we found that in the 14-day-old seedlings, the number of helical roots in the *atpiezo* mutants is significantly higher than the WT (Figure S4A,B). Consistently, we observed that the *atpiezo* mutants showed more lateral roots compared to WT plants in the medium (Figure S4C).

### 2.5. AtPiezo Affects the Shape of Root Cap in Response to Mechanical Forces

**Figure 5:**
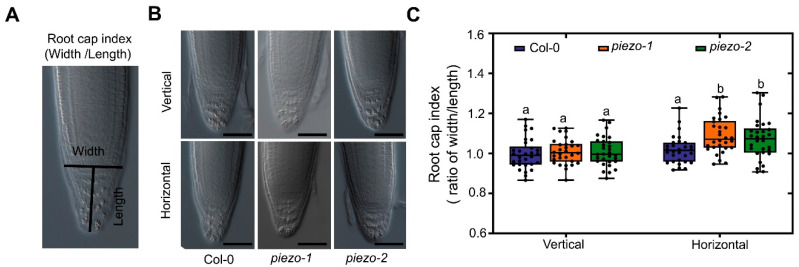
AtPiezo affects the shape of root caps in response to mechanical forces. (**A**) Schematic diagram of root cap index. The index is the ratio of the width across the QC and the length from QC to the root tip. (**B**) Root cap shape of Col-0, *piezo1*, and *piezo-2* grown on the medium with 0.8% agar. The root cap of 4-day-old plants that vertically grew on the agar medium (Vertical). The root cap of the plants that horizontally grew for 24 h followed by vertical growth for three days on the medium (Horizontal). Bar = 50 μm. (**C**) Statistic analysis of root cap index for different plants under vertical and horizontal growth conditions. Boxplots span the first to the third quartiles of the data. A line in the box represents the median (*n* = 30). The results were analyzed with two-way ANOVA and Tukey’s multiple comparison test (Different lowercase letters indicate significant differences at *p* < 0.05).

To further analyze the function of AtPiezo in response to mechanical force at the root cap, we observed the morphology of root cap under different growth conditions by analyzing root cap index (Figure 5A). The root cap index is defined as the ratio of the width across the quiescent center (QC) and the length from QC to the root tip. When the plants grew vertically, there was no significant difference in root cap morphology between WT and *atpiezo* mutants. However, the root cap index of *atpiezo* mutants increased compared to WT when the plants grew on horizontally placed agar medium (Figure 5B,C). We also found root cap morphology of helical roots and non-helical roots are different. The width of the root cap increased in helical root and the root cap morphology of helical roots of *atpiezo* mutants is similar to that in WT plants (Figure S5A,B). We speculate that the root cap of *atpiezo* mutants are less sensitive to mechanical forces, leading to altered root cap morphology which may impact root growth direction and determine root architecture.

### 2.6. AtPiezo Change the Ca^2+^ Flux of Plant Root Cap

**Figure 6:**
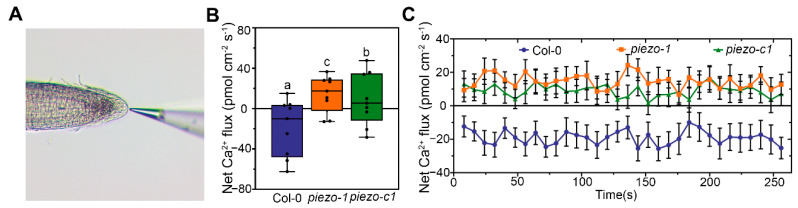
AtPiezo affects the Ca^2+^ flux of plant root cap. (**A**) Schematic diagram of the position for Ca^2+^ flux measurement using NMT. (**B**) The net Ca^2+^ fluxes in Col-0, *piezo-1*, and *piezo-c1* seedlings that were grown on medium for five days. Boxplots span the first to the third quartiles of the data. A line in the box represents the median (*n* = 9). The results were analyzed with one-way ANOVA and Tukey’s multiple comparison test (Different lowercase letters indicate significant differences at *p* < 0.05) (**C**) Real-time kinetics of Ca^2+^ flow in the tip of root cap. Data are presented as mean ± SE (*n* = 5).

In animals, Piezo proteins function as non-selective cation influx ion channels. In particular, Piezo proteins mainly mediate the flux of Ca^2+^ [39]. In *Arabidopsis*, we found that the pattern of Ca^2+^ gradience is similar to the expression of *AtPiezo* gene in the root tip (Figure S6A). We conjectured that AtPiezo may function in mediating the flow of Ca^2+^. To verify this speculation, we crossed the plants harboring a GFP-based Ca^2+^ indicator (GCaMP6s) to the *atpiezo* mutants. Under normal growth conditions, we found that the Ca^2+^ concentration of the mutants did not change obviously (Figure S6B,C). Then we used the non-damaging micro-measurement technique (NMT) to detect the flux of Ca^2+^ in root cap (Figure 6A). In the liquid testing environment, we found that the Net Ca^2+^ influx of WT is obviously higher than that in the *atpiezo* mutants at the root cap (Figure 6B,C). These results suggest that AtPiezo may influence the flow of Ca^2+^ in the root cap.

## 3. Discussion

In this study, we investigated the potential function of the homologous gene of animal *Piezo* gene in plants. By analyzing the expression patterns of *AtPiezo* gene in *Arabidopsis*, we found *AtPiezo* is highly expressed in the root cap. In order to analyze the biological functions of *AtPiezo*, we isolated two T-DNA insertion alleles and one CRISPR/Cas9-mediated genomic deletion line. We found the loss-of-function mutants of *AtPiezo* show impaired rooting ability on the agar medium. In addition, when growing within the agar medium, the *atpiezo* mutants showed increased numbers of helical roots and lateral roots. These results indicated AtPiezo likely plays an important role in sensing mechanical stimuli in root. Moreover, *atpiezo* mutants displayed altered morphology of root tip compared to WT plants, suggesting the function of AtPiezo in both sensing physical force and regulating root development. Furthermore, by using the NMT method, we found calcium influx is reduced in *atpiezo* mutants compared to WT plants, indicating AtPiezo likely functions as a Ca^2+^ channel in plants, similar to its homologs in animals. In summary, our results demonstrated that the homolog of animal Piezo proteins is also functional in plants. The functional analyses on AtPiezo indicated it may play an important role in root cap sensing the mechanical force from the environment when the root is foraging in the soil or medium.

The protein structure analyses of MmPiezo1 indicated that the Piezo proteins contain essential domains including piezo repeats, beam, CAP, inner helix and CTD [31,34,36]. Upon sensing the membrane tension, piezo repeats domain will be deformed. This conformational change will be transmitted via beam domain to CTD domain [36]. CTD eventually mediates the opening of CAP domain via inner helix. CAP, CTD and inner helix domains are essential parts of channel pore structure [36]. Our results indicated that the inner helix and CTD domains in AtPiezo show high similarity to those in animal Piezo protein, suggesting the pore structure is highly conservative in different Piezo proteins. Whereas, the beam domain is less conservative, indicating the force transmission in Piezo proteins likely varies in different species.

The root cap is an important structure that protects the root of plants when growing through the soil and perceives the mechanical force of soil [22]. The growth and development of the root cap are dynamic processes [23]. The cell wall of outermost columella cells will degrade, causing the cell layer to fall off naturally [24]. Our result showed *AtPiezo* has a higher expression in the root cap, especially in the cells processing shedding (Figure 2C). We thus conjectured that cell wall degradation may cause AtPiezo to function in the shedding cells. In this study, we found that AtPiezo plays a role in plant root cap to respond to mechanical force. Previous studies have shown that the shape of root cap will be dramatically altered when growing in different soil status [21,25]. We thus observed the morphology of root cap of the *atpiezo* mutants under different growth conditions. When grown in the medium with 0.8% agar, the *atpiezo* mutant plants showed increased helical roots compared to WT plants. Our result indicated that the morphology of non-helical roots is similar in *atpiezo* mutant and WT plants. Additionally, the morphology of helical roots is largely as same as that in *atpiezo* mutants and WT. We believe that the helical root phenotype is likely the consequence of root morphology. Since horizontally grown *atpiezo* mutant roots have statistically wider root cap in comparison to WT, the roots have a higher possibility to show helical phenotype in *atpiezo* plants. However, once the roots already show helical or non-helical phenotype, the root morphology is likely similar in *atpiezo* mutant and WT plants. Whereas, at the current stage, how AtPiezo affects the shape of the root cap in response to mechanical stimuli is still unknown.

A recent report showed that when *Arabidopsis* plants are grown in agar medium, the growth force and external mechanical forces (e.g., the resistance from agar media) together contribute to the phenotype of helical roots [35]. The increased helical roots in *atpiezo* mutant plants may be caused by their reduced ability to respond to external mechanical forces. Furthermore, it remains to be clarified whether AtPiezo also senses internal mechanical signals such as growth force.

Serving as an important second messenger, Ca^2+^ has been reported to participate in the processes of signal transmission in response to various environmental stimuli [40]. Studies have shown that Ca^2+^ plays an indispensable role in the root responses to mechanical damage, obstacles and the changes in turgor pressure [41]. MmPiezo1 acts as a non-selective cation inflow ion channel in mouse, as Ca^2+^ is the major ion [39]. Due to the conservativeness of the ion pore structure, we speculate that AtPiezo is also likely to mediate the Ca^2+^ flux in plants. This is indirectly verified by the results of Ca^2+^ flow in the root cap of *atpiezo* mutants. Future electrophysiological analysis will provide solid evidence to show whether AtPiezo indeed functions as a Ca^2+^ channel.

Based on our results, we believe that AtPiezo plays an important role in response to mechanical forces in root. As a potential cation channel, AtPiezo may influence the Ca^2+^ transportation upon different mechanical stimuli. Ca^2+^ regulates multiple downstream biological events in root, such as the growth and development of root tip, and root cap morphology. Loss of *AtPiezo* gene leads to disrupted responses of plants to mechanical forces in root.

To further determine the function of AtPiezo, we set to clone the CDS sequence of the *AtPiezo* gene that is 7455 bp. After numerous attempts, we successfully constructed a plant expression vector in a special bacterial strain EPI400. However, the expression vector of *AtPiezo* gene cannot exist in Agrobacterium. Therefore, we have not yet obtained the genetically modified lines of *AtPiezo* gene.

*AtPiezo* is not only highly expressed in root cap but also in vascular tissue, guard cell, and pollen. Further studies will be focused on the investigations of the roles of *AtPiezo* in these tissues. Our current study of *AtPiezo* revealed the similarities and differences of the roles of Piezo between plants and animals. It provided us better understanding of the processes of plants in response to mechanical force.

## 4. Materials and Methods

### 4.1. Phylogenetic and Sequences Analysis

All the protein sequences of putative MmPiezo1 homologs in the selected 37 representative species were identified by BLAST using NCBI [42] (https://www.ncbi.nlm.nih.gov/) and UniProt [43] (https://www.uniprot.org/). Only one was selected when multiple isoforms of Piezo homolog were found. The sequence information was listed in Table S1. Phylogenetic analysis was performed with MEGA7 software [44] (Temple University, Philadelphia, PA, USA). For the tree depicted in Figure 1A and Figure S1A, 60 full-length protein sequences were aligned using MUSCLE method with default settings. For the Maximum Likelihood tree, Poisson model was used with 50% partial deletion for Gaps Data Treatment set. The phylogeny test used 500 bootstrap replications. For the Neighbor-Joining tree, Poisson model was used with 50% partial deletion for Gaps Data Treatment set. The phylogeny test used 1000 bootstrap replications. The TMHMM Server v.2.0 [45] (http://www.cbs.dtu.dk/services/TMHMM/), Phobius [46] (http://phobius.sbc.su.se/), SMART [47] (http://smart.embl-heidelberg.de/) was used to predict the transmembrane domains and functional domain. For visualization of proteoform, an online tool Protter [48] (http://wlab.ethz.ch/protter/start/) was used. Multiple sequence alignment was performed with SnapGene v.4.2.4 software (GSL Biotech LLC, Chicago, IL, USA) and the application used Clustal Omega method with default settings. The picture of *Piezo* homologs gain or loss tree in plant kingdom was downloaded from the Ensembl Plants online database [49] (http://plants.ensembl.org/).

### 4.2. Plant Materials and Growth Conditions

*Arabidopsis thaliana* Columbia accession (Col-0) was used as wild-type, and the T-DNA insertion alleles *piezo-1* (SALK_003005), *piezo-2* (SAIL_856_B11) in Col-0 background were obtained from the *Arabidopsis* Biological Resource Center (ABRC). The *piezo-c1* mutant in Col-0 background was generated using a CRISPR/Cas9 system from professor Qijun Chen (Huazhong Agricultural University, Wuhan, China). The method as was described previously [50]. For transgenic plants, plants were transformed using the Agrobacterium tumefaciens-mediated floral dip method [51]. *Arabidopsis* were soil-grown in a greenhouse under long-day light conditions (16 h light/8 h dark per day, light intensity, 22 ± 2 °C) In phenotypic analysis, surface-sterilized *Arabidopsis* seeds were placed at 4 °C for 48 h and then geminated vertically or horizontal on half-strength Murashige and Skoog (1/2 MS) (Phytotech, Lenexa, KS, USA), 1% sucrose (*w*/*v*) with different concentrations of agar (Solarbio, Beijing, China). Plates were placed in a growth chamber at 22 °C with 16 h light/8 h dark condition.

### 4.3. Genotyping Atpiezo Mutants and Cloning AtPiezo

To genotype the mutants, genomic DNA and RNA were extracted from the mutant leaves, then PCR and RT-PCR were performed with gene-specific primers as listed in Supplementary Table S2. The 7455bp sequence of the *AtPiezo* gene without stop codon was cloned from the cDNA of Col-0. The *AtPiezo* gene was cloned into a plant expression vector *pBIB-Basta-35S-YFP*, using cloning sites of *Kpn*Ⅰ and *Xba*Ⅰ. EPI400 strain (GENEWIZ, Suzhou, China) was used to propagate vectors.

### 4.4. Plasmid Construction

To construct the *pAtPiezo::GUS* and *pAtPiezo::NLS-YFP*, a 1559bp upstream sequence from *AtPiezo* start codon was amplified from Col-0 genomic DNA, and cloned into the *pDONR/Zeo* vector (Invitrogen, Carlsbad, CA, USA). The entry clones were then used to transfer promoter sequences into a destination vectors of *pBIB-Basta-GWR-GUS* and *pFYTAG* [52] for plant expression.

### 4.5. Phenotypic Analysis of Root Growth

Plants were grown on a horizontally placed medium for four days in the experiment of rooting ability analyze. The rooting ratio of plants was determined by observing whether the root tips of plants were in the medium. To analyze helical roots, plants were grown vertically in medium with different concentrations of agar. Photos were taken with a digital camera (Canon 450D, Canon, Tokyo, Japan). To clearly visualize the root growth pattern in agar media, the roots of 14-day-old plants were observed using a Leica microscope. To analyze the index of root cap, the photos of the roots grown on different media for four days were taken using Fluorescence microscope (ZEISS Axio Imager.Z2, ZEISS, Oberkochen, Germany). The root cap indexes were determined by measuring the width across the QC and the length from QC to the root tip (ImageJ v.1.52p, NIH, Bethesda, MD, USA).

### 4.6. GUS Staining and Cross Sectioning

Different developmental stages and different tissues of transgenic plants were collected and stained. Tissues were first incubated in rinse solution (34.2 mM Na_2_HPO_4_, 15.8 mM NaH_2_PO_4_, 0.5 mM K_3_Fe(CN)_6_, and 0.5 mM K_4_Fe(CN)_6_.3H_2_O) for 5 min, then incubated in stain solution (34.2 mM Na_2_HPO_4_, 15.8 mM NaH_2_PO_4_, 0.5 mM K_3_Fe(CN)_6_, 0.5 mM K_4_Fe(CN)_6_.3H_2_O, and 2 mM X-Gluc) at 37 °C for appropriate time. After GUS staining, the plant tissues were immersed in 30%, 50%, 75% and 95% ethanol for 1 h in turn, and then immersed in 75% ethanol. After being decolorized, the tissues were observed with the stereomicroscope (Leica M165 C, Leica, Wetzlar, Germany). For root cross sectioning, the GUS stained 4-days-old seedling were fixed in FAA fixer (50% (*v*/*v*) ethanol, 5% (*v*/*v*) acetate acid, and 3.7% (*v*/*v*) formaldehyde), embedded in Technovit 7100 resin, and then sectioned with a Leica microtome (RM2245, Leica, Wetzlar, Germany). Different layers of the root were sectioned at 5 µm thickness. Placed on coverslips to observed and took pictures with fluorescence microscope (ZEISS, Axio Imager.Z2, ZEISS, Oberkochen, Germany).

### 4.7. In Vivo Ca^2+^ Imaging

Transgenic *Arabidopsis* plants stably expressing GCaMP6s were used for in vivo imaging as previously described [53]. In brief, 4-day-old seedling was loaded onto the slide and cytoplasmic Ca^2+^ imaging was obtained using ZEISS laser scanning confocal system (ZEISS LSM 880 AxioObserver, ZEISS, Oberkochen, Germany). The excitation was provided at 488 nm and images were collected at emission 493–598 nm. The scanning resolution was set at 1024 × 1024 pixels with the 20× objective lens. GCaMP6s signals were analyzed by using ZEN 3.1 (blue edition) software (ZEISS, Oberkochen, Germany).

### 4.8. Measurement of the Net Ca^2+^ Fluxes in Root Cap

The net fluxes of Ca^2+^ were measured non-invasively using the NMT (Non-invasive Micro-test Technology) with NMS (Non-invasive Micro-test system) (NMT150S, Younger USA LLC, Amherst, MA, USA) and in Fluxes V2.0 (Younger USA LLC, Amherst, MA, USA) software. The microelectrode, LIX Holder, Ca^2+^-LIX, and Ag/AgCl wire microsensor holder used in the experiment were purchased from Xuyue (Beijing) Science and Technology Co., Ltd., Beijing, China. The universal method was described in previous reports [54]. In this study, plants were grown on medium for 4 days, then transferred to the measuring solution (0.1 mM KCl, 0.1 mM CaCl_2_, 0.1 mM MgCl_2_, 0.5 mM NaCl, 0.3 mM MES, 0.2 mM Na_2_SO_4_, pH = 6.0) for measurement. Calibration solution 1 (0.1 mM KCl, 0.02 mM CaCl_2_, 0.1 mM MgCl_2_, 0.5 mM NaCl, 0.3 mM MES, 0.2 mM Na_2_SO_4_, pH = 6.0) and the calibration solution 2 (0.1 mM KCl, 1 mM CaCl_2_, 0.1 mM MgCl_2_, 0.5 mM NaCl, 0.3 mM MES, 0.2 mM Na_2_SO_4_, pH = 6.0) were used for correction. Ion flux recordings were taken for at least 8 min for each plant root cap and the measured data were rejected during the first 1 min. At least 5 plants were measured.

### 4.9. Data Acquisition and Statistics

We used the image software ImageJ.v.1.52p (NIH, Bethesda, MD, USA)to measure the original picture. All experiments were repeated at least three times. The Statistical analyses were indicated in each figure legend and were performed using the program GraphPad Prism.v.8 (GraphPad Software, San Diego, CA, USA). Statistical significance is indicated by the *p* value, *p* < 0.05, marked with different characters.

## Data Availability

The T-DNA inserted mutants *piezo-1* (SALK_003005) and *piezo-2* (SAIL_856_B11) in Col-0 background were obtained from the Arabidopsis Biological Resource Center (ABRC, https://abrc.osu.edu/).

## Supplementary Materials

Supplementary Materials can be found at https://www.mdpi.com/1422-0067/22/1/467/s1. Figure S1. Piezo phylogenetic Tree and gene gain or loss tree. Figure S2. Representative images of the expression pattern of *AtPiezo* in *Arabidopsis*. Figure S3. Isolation of *atpiezo* mutants. Figure S4. AtPiezo affects the root architecture in the medium. Figure S5. Root cap shape of the root foraging in the medium. Figure S6. Ca^2+^ gradience in the root cap. Table S1. Information of protein sequences in phylogenetic analysis. Table S2. Primer sequences used in this study.
