# Supplementary material for: AtPiezo Plays an Important Role in Root Cap Mechanotransduction
Source: Int J Mol Sci. 2021 Jan 5;22(1):467. doi: 10.3390/ijms22010467 (PMC7796506; doi:10.3390/ijms22010467)
Supplement: Supplementary file 1 [file ijms-22-00467-s001.pdf]

International Journal of *Molecular Sciences*

MDPI

*Supplementary*

# AtPiezo plays an important role in root cap mechanotransduction

**Xianming Fang [1], Beibei Liu [1], Qianshuo Shao [1], Xuemei Huang [1], Jia Li [1], Sheng Luan [2,*] and Kai He [1,*]**

[1] Ministry of Education Key Laboratory of Cell Activities and Stress Adaptations, School of Life Sciences, Lanzhou University, Lanzhou 730000, China; fangxm18@lzu.edu.cn (X.F.); liubb19@lzu.edu.cn (B.L.); shaoqsh19@lzu.edu.cn (Q.S.); huangxm17@lzu.edu.cn (X.H.); lijia@lzu.edu.cn (J.L.)

[2] Department of Plant and Microbial Biology, University of California, Berkeley, California 94720, USA

* Correspondence: hekai@lzu.edu.cn (K.H.); sluan@berkeley.edu (S.L.)

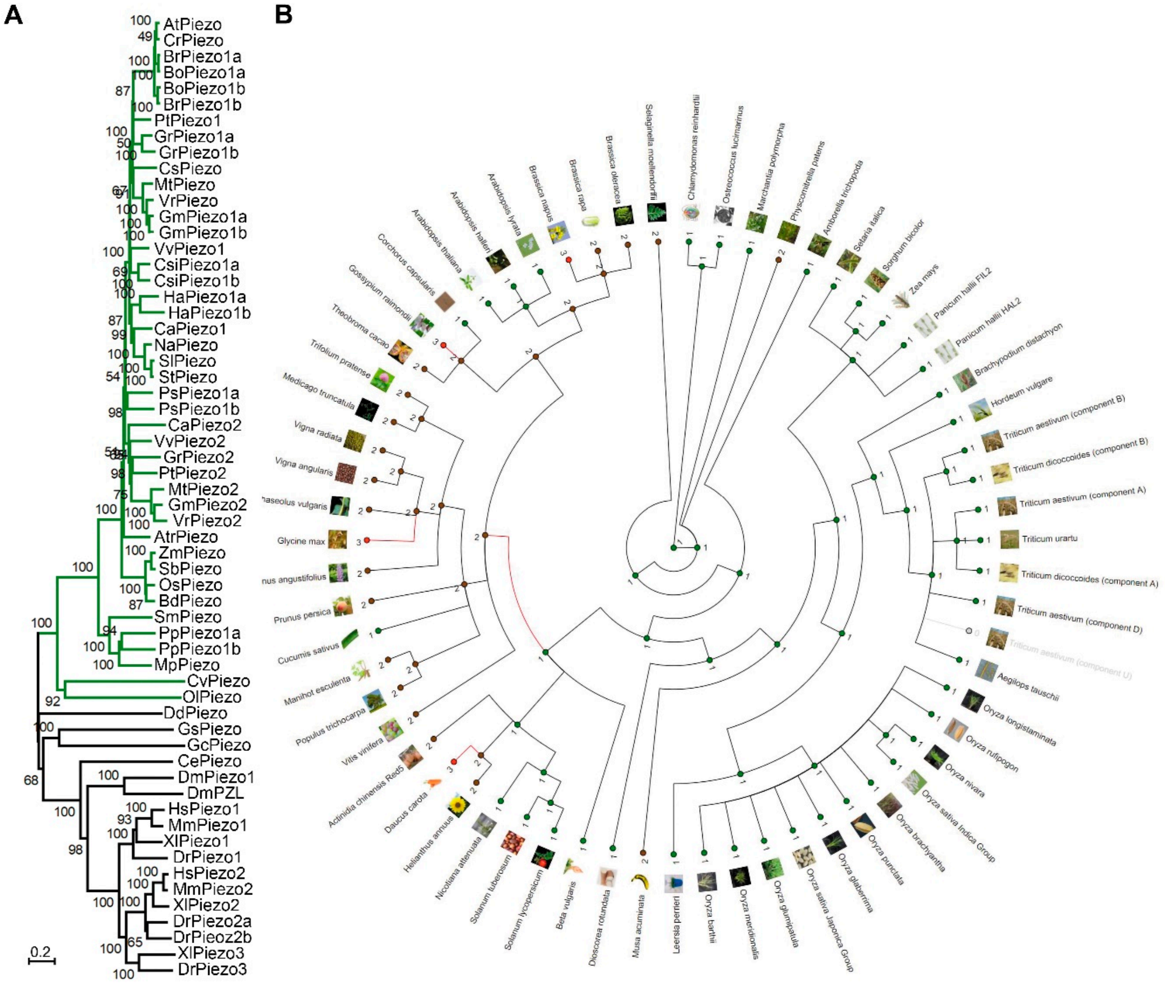


**Figure S1.** Piezo phylogenetic Tree and gene gain or loss tree. (**A**) Neighbor-Joining Unrooted Phylogenetic Tree of Piezo Homologs. Green branches belong to the plant kingdom. (**B**) Piezo homologs gain or loss tree in plant kingdom.

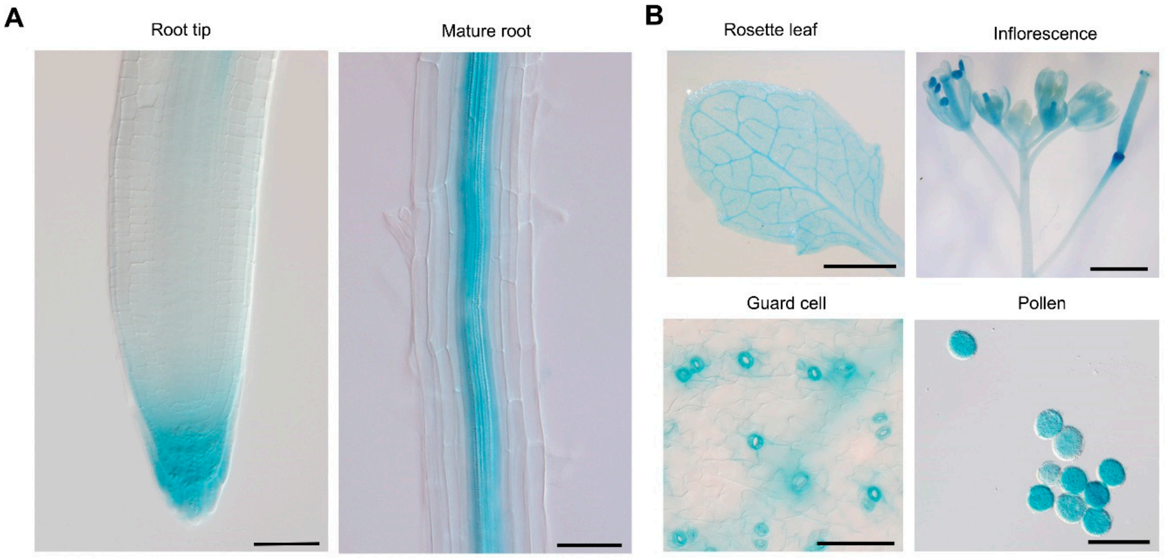


**Figure S2.** Representative images of the expression pattern of *AtPiezo* in *Arabidopsis*. (**A**) Histochemical GUS staining is shown in root tip and mature zone of primary root of 4-day-old seedling. Bars= 50μm. (**B**) Expression patterns of *pAtPiezo::GUS* in different organs. Histochemical GUS staining is shown in rosette leaf, guard cell, inflorescence and pollen. Bars=1mm (rosette leaf), 100μm (guard cell), 2mm (inflorescence), and 50μm (pollen).

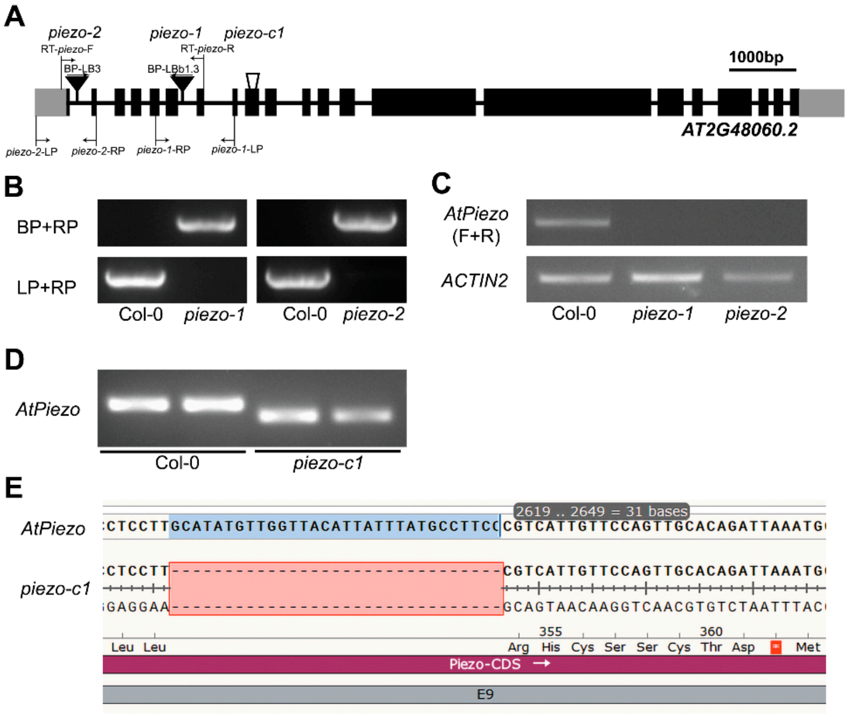


**Figure S3.** Isolation of *atpiezo* mutants. (**A**) Schematic diagram of *AtPiezo* gene structure with the localization of T-DNA insertions and primers used for mutant identification (**B**) T-DNA insertion in *piezo-1* and *piezo-2* was determined by PCR. (**C**) RT-PCR confirmed *piezo-1* and *piezo-2* are null alleles. *ACTIN2* was used as a positive control. (**D**) Deletion of genomic DNA fragment in *piezo-c1* mutant was determined by PCR. (**E**). *piezo-c1* has a 31-bp deletion in the genomic DNA of *AtPiezo*.

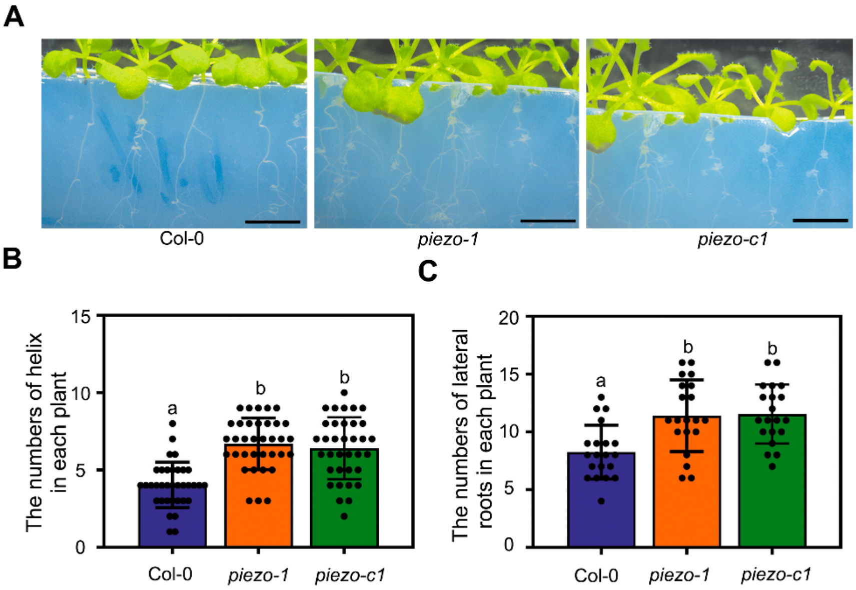


**Figure S4**. AtPiezo affects the root architecture in the medium. (**A**) Representative images of root growth status of different plants in the medium. 14-day-old seedlings of WT and *atpiezo* mutants were grown in the medium with 0.8% agar. Bar=1cm. (**B**) Statistic analysis of the helical roots in 14-day-old seedlings of WT and *atpiezo* mutants. Data are presented as mean ± SD (n ⩾ 30). The results were analyzed with one-way ANOVA and Tukey's multiple comparison test (Different lowercase letters indicate significant differences at $p < 0.05$). (**C**) Statistics analysis of the lateral roots in 14-day-old seedlings of WT and *atpiezo* mutants. Data are presented as mean ± SD (n ⩾ 30). The results were analyzed with two-way ANOVA and Tukey's multiple comparison test (Different lowercase letters indicate significant differences at $p < 0.05$).

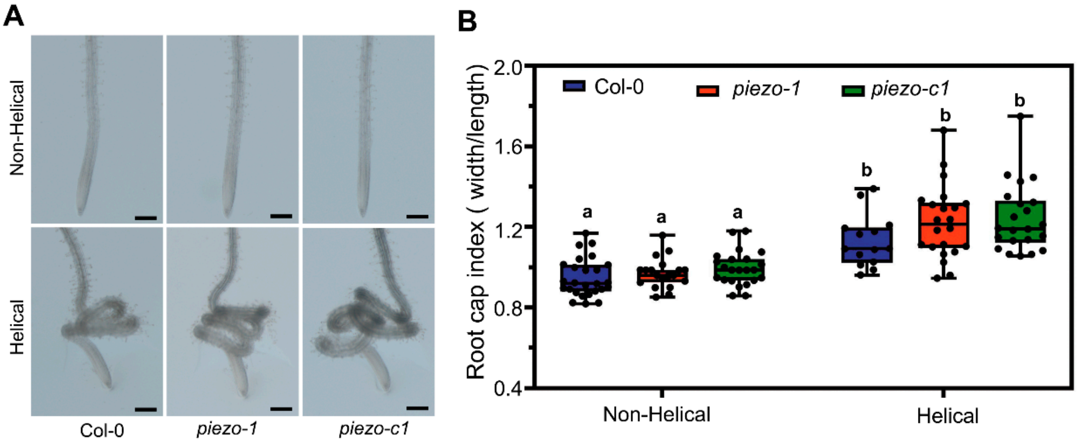


**Figure S5.** Root cap shape of the root foraging in the medium. (**A**) The root cap shape of WT and *atpiezo* mutants in the agar medium. Four-day-old seedlings were grown in medium with 0.8% agar. Bar=0.2mm. (**B**) Statistic analysis of root cap index for helical and non-helical roots of WT and *atpiezo* mutants in medium. Boxplots span the first to the third quartiles of the data. A line in the box represents the median ($n \geqslant 14$). The results were analyzed with two-way ANOVA and Tukey's multiple comparison test (Different lowercase letters indicate significant differences at $p < 0.05$).

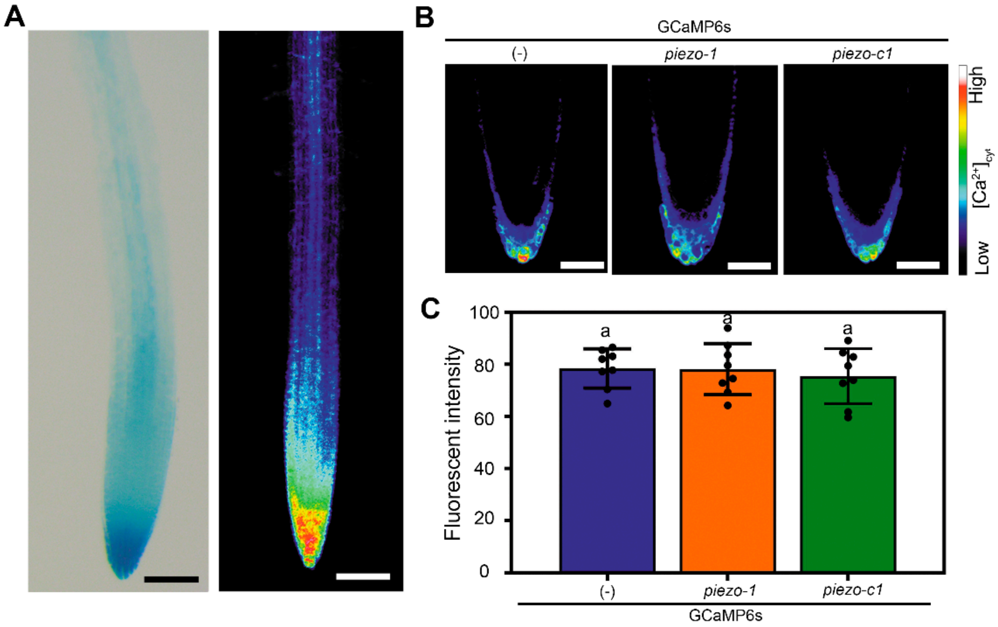


**Figure S6.** $Ca^{2+}$ gradience in the root cap. (**A**) The distribution of $Ca^{2+}$ is similar to the expression pattern of *AtPiezo* gene in the root cap. Histochemical GUS staining is shown in 4-days-old seedling of *pAtPiezo::GUS* lines (left). $Ca^{2+}$ signal was detected in the root cap of WT plants harboring GCaMP6s (right). Bar=100μm. (**B**) $Ca^{2+}$ signal in root caps of WT and the *atpiezo* mutants. Bar=50μm. (**C**) Fluorescent intensity of $Ca^{2+}$ in WT and *atpiezo* mutants. Data are presented as mean ± SD ($n = 8$). The results were analyzed with one-way ANOVA and Tukey's multiple comparison test (Different lowercase letters indicate significant differences at $p < 0.05$).

**Table S1. Information of protein sequences in phylogenetic analysis.**

| No. | Species | Protein name | Predicted Size (aa) | Sequence identifies |
|---|---|---|---|---|
| 1 | *Homo sapiens* | **HsPiezo1** | 2521 | NP_001136336.2 |
| 2 | *Homo sapiens* | **HsPiezo2** | 2752 | NP_071351.2 |
| 3 | *Mus musculus* | **MmPiezo1** | 2546 | NP_001032375.1 |
| 4 | *Mus musculus* | **MmPiezo2** | 2824 | NP_001034574.4 |
| 5 | *Xenopus laevis* | **XlPiezo1** | 2475 | XP_018096992.1 |
| 6 | *Xenopus laevis* | **XlPiezo2** | 2830 | XP_018123363.1 |
| 7 | *Xenopus laevis* | **XlPiezo3** | 2776 | XP_018092291.1 |
| 8 | *Danio rerio* | **DrPiezo1** | 2538 | XP_696355.4 |
| 9 | *Danio rerio* | **DrPiezo2a** | 2752 | XP_021325930.1 |
| 10 | *Danio rerio* | **DrPiezo2b** | 3005 | XP_021323945.1 |
| 11 | *Danio rerio* | **DrPiezo3** | 2634 | XP_021334475.1 |
| 12 | *Drosophila melanogaster* | **DmPiezo1** | 2533 | NP_001188719.1 |
| 13 | *Drosophila melanogaster* | **DmPiezo2（Pzl）** | 2326 | NP_001303493.1 |
| 14 | *Caenorhabditis elegans* | **CePiezo** | 2402 | NP_001293979.1 |
| 15 | *Dictyostelium discoideum* | **DdPiezo** | 3080 | XP_640187.1 |
| 16 | *Galdieria sulphuraria* | **GsPiezo** | 2823 | XP_005703632.1 |
| 17 | *Chondrus crispus* | **CcPiezo** | 2929 | XP_005712062.1 |
| 18 | *Chlorella variabilis* | **CvPiezo** | 2946 | XP_005847868.1 |
| 19 | *Ostreococcus lucimarinus* | **OlPiezo** | 2372 | XP_001418409.1 |
| 20 | *Marchantia polymorpha* | **MpPiezo** | 2626 | A0A2R6XCN6 |
| 21 | *Physcomitrella patens* | **PpPiezo1a** | 2575 | XP_024385645.1 |
| 22 | *Physcomitrella patens* | **PpPiezo1b** | 2572 | XP_024369685.1 |
| 23 | *Selaginella moellendorfi* | **SmPiezo** | 2399 | XP_024535480.1 |
| 24 | *Zea mays* | **ZmPiezo** | 2505 | XP_020405822.1 |
| 25 | *Sorghum bicolor* | **SbPiezo** | 2504 | XP_021311180.1 |
| 26 | *Oryza sativa Japonica Group* | **OsPiezo** | 2557 | XP_025881504.1 |
| 27 | *Brachypodium distachyon* | **BdPiezo** | 2503 | XP_010230893.1 |
| 28 | *Amborella trichopoda* | **AtrPiezo** | 2485 | XP_020526919.1 |
| 29 | *Medicago truncatula* | **MtPiezo1** | 2478 | XP_024639854.1 |
| 30 | *Medicago truncatula* | **MtPiezo2** | 2462 | XP_003626328.2 |
| 31 | *Glycine max* | **GmPiezo1a** | 2483 | XP_014618394.1 |
| 32 | *Glycine max* | **GmPiezo1b** | 2482 | XP_006575092.1 |
| 33 | *Glycine max* | **GmPiezo2** | 2310 | XP_025982938.1 |
| 34 | *Vigna radiata* | **VrPiezo1** | 2465 | XP_022642325.1 |
| 35 | *Vigna radiata* | **VrPiezo2** | 2483 | XP_022640429.1 |
| 36 | *Vitis vinifera* | **VvPiezo1** | 2487 | XP_019076639.1 |
| 37 | *Vitis vinifera* | **VvPiezo2** | 2481 | XP_010660075.1 |
| 38 | *Gossypium raimondii* | **GrPiezo1a** | 2478 | XP_012476188.1 |
| 39 | *Gossypium raimondii* | **GrPiezo1b** | 2476 | XP_012490496.1 |

| | | | | |
|---|---|---|---|---|
| 40 | *Gossypium raimondii* | **GrPiezo2** | 2469 | XP_012462417.1 |
| 41 | *Coffea arabica* | **CaPiezo1** | 2475 | XP_027116044.1 |
| 42 | *Coffea arabica* | **CaPiezo1** | 2468 | XP_027114534.1 |
| 43 | *Populus trichocarpa* | **PtPiezo1** | 2482 | XP_024440850.1 |
| 44 | *Populus trichocarpa* | **PtPiezo2** | 2461 | XP_024456948.1 |
| 45 | *Nicotiana attenuata* | **NaPiezo** | 2473 | XP_019228329.1 |
| 46 | *Helianthus annuus* | **HaPiezo1a** | 2441 | XP_022035444.1 |
| 47 | *Helianthus annuus* | **HaPiezo1b** | 2480 | XP_022010653.1 |
| 48 | *Cucumis sativus* | **CsPiezo1** | 2438 | XP_011659324.1 |
| 49 | *Solanum lycopersicum* | **SlPiezo1** | 2473 | XP_010326620.1 |
| 50 | *Solanum tuberosum* | **StPiezo1** | 2473 | XP_006358438.1 |
| 51 | *Camellia sinensis* | **CsiPiezo1a** | 2468 | XP_028067352.1 |
| 52 | *Camellia sinensis* | **CsiPiezo1b** | 2489 | XP_028090451.1 |
| 53 | *Brassica oleracea* | **BoPiezo1a** | 2481 | XP_013635988.1 |
| 54 | *Brassica oleracea* | **BoPiezo1b** | 2485 | XP_013635938.1 |
| 55 | *Brassica rapa* | **BrPiezo1b** | 2485 | XP_009142485.1 |
| 56 | *Brassica rapa* | **BrPiezo1a** | 2482 | XP_009118144.2 |
| 57 | *Capsella rubella* | **CrPiezo** | 2485 | XP_006293550.1 |
| 58 | *Arabidopsis thaliana* | **AtPiezo** | 2485 | NP_001323909.1 |
| 59 | *Papaver somniferum* | **PsPiezo1** | 2485 | XP_026456484.1 |
| 60 | *Papaver somniferum* | **PsPiezo2** | 2480 | XP_026450010.1 |

**Table S2. Primer sequences used in this study.**

| Primer Name | Sequence (5'→3') |
|---|---|
| RT-*ACTIN2*-F | TGCTGTGATTTCTTTGCTCATACG |
| RT-*ACTIN2*-R | CAGTGGTCGTACAACCGGTATTG |
| RT-*AtPiezo*-F | AGCAGAATCCCCTCCGTACT |
| RT-*AtPiezo*-R | ATGGCCTGGAGAGCTAGTGA |
| BP-LBb1.3 | ATTTTGCCGATTTCGGAAC |
| BP-LB3 | TAGCATCTGAATTTCATAACCAATCTCGATACAC |
| *piezo-1*-LP | CCCCTCAGTAAAACATTGGTG |
| *piezo-1*-RP | TCTTGTGGACTGGTCCTTGAC |
| *piezo-2*-LP | GTCCTTGGGTTCTAATCCGTC |
| *piezo-2*-RP | CAGGCTTACCAATTTCAGGTG |
| RT-*piezo*-F | CTTTTGGAAGAAATTGAGGTCG |
| RT-*piezo*-R | CTTGATTCTCGAATAAAAAATG |
| Cas9-*piezo-c1*-T1-BsF | ATATATGGTCTCGATTGAACTGGAACAATGACGGGAGTT |
| Cas9-*piezo-c1*-T1-F0 | TGAACTGGAACAATGACGGGAGTTTTAGAGCTAGAAATAGC |
| Cas9-*piezo-c1*-T2-R0 | AACCCTTGCATATGTTGGTTACCAATCTCTTAGTCGACTCTAC |
| Cas9-*piezo-c1*-T2-BsR | ATTATTGGTCTCGAAACCCTTGCATATGTTGGTTACCAA |
| Identify-*piezo-c1*-F | TATTTGCGTTCATGATTGCCAG |
| Identify-*piezo-c1*-R | AGGCCACATTGAATATATACGT |
| Promoter-*AtPiezo*-F | AAAAAGCAGGCTTCTACAAGAAATATTCCTTAAGG |
| Promoter-*AtPiezo*-R | AGAAAGCTGGGTCTGGAAACTTTTGTCTTAACGA |
| Infusion-Kpn Ⅰ -*Piezo*CDS-F | TTAAACTTAAGCTTGGTACCATGGCGAGTTTTTTGGTGGGCT |
| Infusion-Xba Ⅰ -*Piezo*CDS-R | AAACGGGCCCTCTAGAAGCATCATAGTCTAGCTTTGTAT |

**Publisher's Note:** MDPI stays neutral with regard to jurisdictional claims in published maps and institutional affiliations.

© 2020 by the authors. Submitted for possible open access publication under the terms and conditions of the Creative Commons Attribution (CC BY) license (http://creativecommons.org/licenses/by/4.0/).
